# Comparative transcriptome analysis within the Lolium/Festuca species complex reveals high sequence conservation

**DOI:** 10.1186/s12864-015-1447-y

**Published:** 2015-03-28

**Authors:** Adrian Czaban, Sapna Sharma, Stephen L Byrne, Manuel Spannagl, Klaus FX Mayer, Torben Asp

**Affiliations:** Department of Molecular Biology and Genetics, Aarhus University, Forsøgsvej 1, Slagelse, 4200 Denmark; Plant Genome and Systems Biology, Helmholtz Zentrum München, German Research Center for Environmental Health, Ingolstädter Landstrasse 1, Neuherberg, 85764 Germany

**Keywords:** *Lolium-Festuca* complex, RNAseq, Comparative transcriptomics, Gene families

## Abstract

**Background:**

The *Lolium-Festuca* complex incorporates species from the *Lolium* genera and the broad leaf fescues, both belonging to the subfamily *Pooideae*. This subfamily also includes wheat, barley, oat and rye, making it extremely important to world agriculture. Species within the *Lolium-Festuca* complex show very diverse phenotypes, and many of them are related to agronomically important traits. Analysis of sequenced transcriptomes of these non-model species may shed light on the molecular mechanisms underlying this phenotypic diversity.

**Results:**

We have generated *de novo* transcriptome assemblies for four species from the *Lolium-Festuca* complex, ranging from 52,166 to 72,133 transcripts per assembly. We have also predicted a set of proteins and validated it with a high-confidence protein database from three closely related species (*H. vulgare, B. distachyon* and *O. sativa*). We have obtained gene family clusters for the four species using OrthoMCL and analyzed their inferred phylogenetic relationships. Our results indicate that VRN2 is a candidate gene for differentiating vernalization and non-vernalization types in the *Lolium-Festuca* complex. Grouping of the gene families based on their BLAST identity enabled us to divide ortholog groups into those that are very conserved and those that are more evolutionarily relaxed. The ratio of the non-synonumous to synonymous substitutions enabled us to pinpoint protein sequences evolving in response to positive selection. These proteins may explain some of the differences between the more stress tolerant *Festuca*, and the less stress tolerant *Lolium* species.

**Conclusions:**

Our data presents a comprehensive transcriptome sequence comparison between species from the *Lolium-Festuca* complex, with the identification of potential candidate genes underlying some important phenotypical differences within the complex (such as VRN2). The orthologous genes between the species have a very high %id (91,61%) and the majority of gene families were shared for all of them. It is likely that the knowledge of the genomes will be largely transferable between species within the complex.

**Electronic supplementary material:**

The online version of this article (doi:10.1186/s12864-015-1447-y) contains supplementary material, which is available to authorized users.

## Background

Next Generation Sequencing is a valuable tool for the analysis and study of transcriptomes of non model species [1], especially when resources are limited and a complete re-sequencing of the genome is not practical. Transcriptome sequencing allows us to overcome some of the challenges associated with sequencing complex, highly repetitive and large plant genomes.

The *Lolium-Festuca* complex is a common name for the grasses belonging to both the *Lolium* genus and broad leaved fescues from the *Schedonorus* subgenus of *Festuca*. Both genus are part of the *Poaceae* family [2], but their exact taxonomic relationship is unclear, with reports of a shared common ancestor [3,4] or the *Lolium* diverging from *Festuca* around 2 million years ago [5]. The *Poaceae* family also includes species such as wheat, barley, bamboo, rice, sorghum and sugarcane making it one of the most important plant families from an agricultural, economic and ecological point of view [6]. The *Lolium* genus contains ten species [7] all of which are exclusively diploid in nature [8], whereas the *Festuca* genus comprises 600 species and the ploidy numbers range from diploid up to dodecaploid [9]. The species belonging to the *Lolium-Festuca* complex are thought to be closely related and interspecific crosses between some of them occur naturally in the wild. In fact, Festuloliums, which are a cross between Loliums and Fescues are very well established as agriculturally important plants [10-12]. This has led to many discussions as to the exact taxonomy of the complex, as one can find over 500 names for the few *Lolium* species [13]. However, despite such a close relationship and being universally distributed around the globe, the plants within the complex exhibit significant diversity for agriculturally important traits [14] such as growth speed, root length, forage quality, resistance to biotic and abiotic stresses, annuality and perenniality. The *Lolium* species generally have a good nutrient content and are highly palatable [15]. *L.perenne* can withstand heavy grazing, and *L. multiflorum* is characterized by rapid establishment [16]. All these traits make them a very good choice for animal fodder. On the other hand, *F. pratensis* exhibits higher persistence, with a better developed root system allowing it to grow on lower quality soils. It also exhibits resistance to extreme abiotic conditions, such as drought and cold stress, being found as far north as within the Arctic Circle. Introgression of specific traits within the complex are possible and natural hydrids can be found in north-western Europe [17].

Numerous studies have succesfully introduced important traits from *F. pratensis* into *Loliums*, including crown rust resistance in *L. perenne* [18] and *L. multiflorum* [19-21], freezing tolerance [22] and drought tolerance in *L. multiflorum* [23]. Species from the *Lolium-Festuca* complex provide a large pool of genetic variation, both within single species, as well as within the complex. This makes it possible to breed forage and turf vartieties suited for use under a range of environments. Not suprisingly, *Poacea*, to which both *Festuca* and *Lolium* belong has been proposed as a model clade for comparative genomics [24]. Currently well-annotated genomes for this clade are *Brachypodium distachyon* [25], and *Oryza sativa* [26] with ongoing research into *Hordeum vulgare* [27].

The aims of the study were to; (1) reconstruct the transcriptomes of four species within the *Lolium-Festuca* complex: *F. pratensis, L. multiforum, L. m. westerwoldicum* and *L. temulentum*. This would complement the already published *Lolium perenne* transcriptome [28,29], (2) establish the phylogeny of the species based on orthologous protein sequences, (3) identify and compare gene families across the analyzed transcriptomes, and (4) identify genes under positive selection between the very resistant to biotic and abiotic stresses *F. pratensis*, and more susceptible *Lolium* species.

## Results and discussion

### *De-novo* assembly of transcriptomes from the *Lolium-Festuca* complex

**Figure 1:**
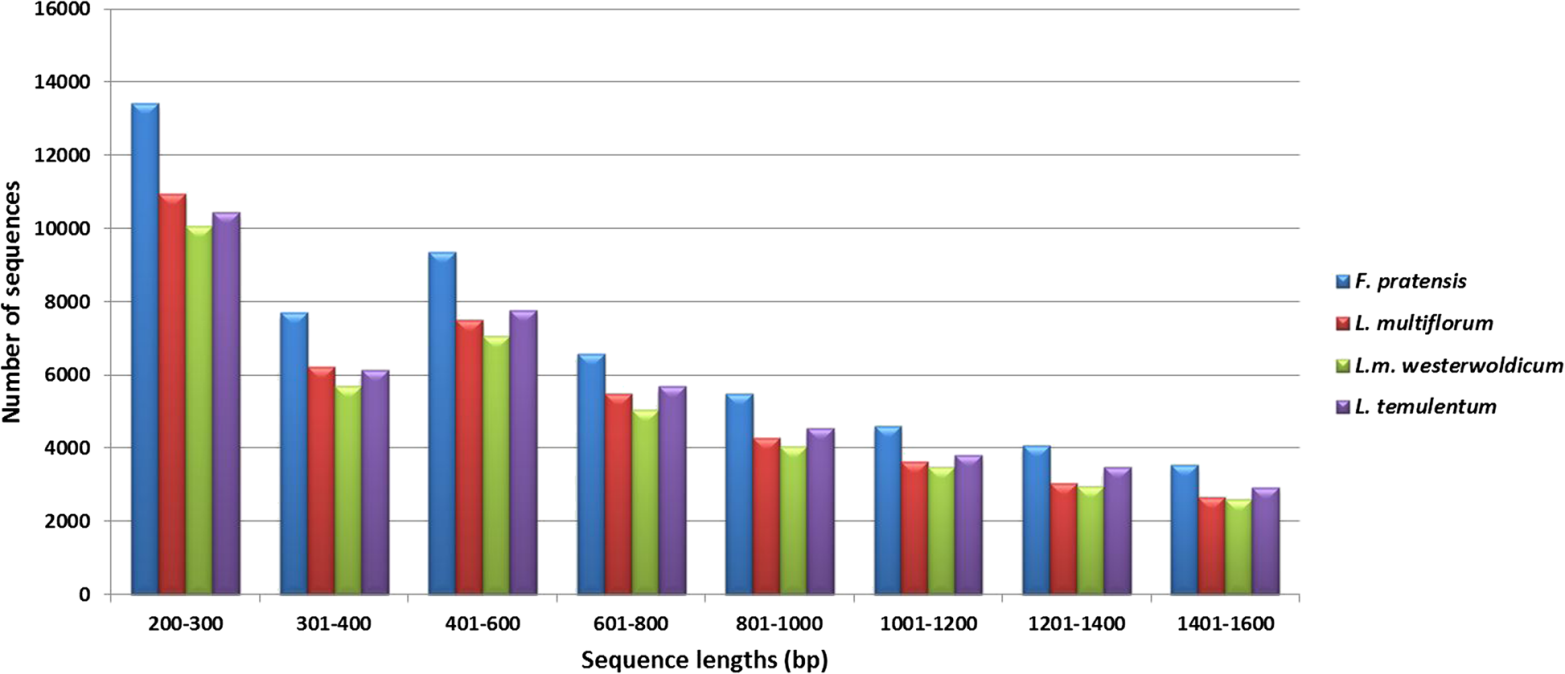
**Length distribution graph.** A vertical bar chart of length distribution of transcriptome assembly fragments across analyzed species. The X-axis represents the length range bins, the Y-axis is the amount of transcripts present in each bin.

We focused on generating transcriptome assemblies for four species within the *Lolium-Festuca* complex. Reads were error-corrected using ALLPATHS-LG tool [30], and assembled using Trinity software [31] to produce transcriptome assemblies that varied in transcript number between 52,166 and 72,133 after quality filtering for low-read support transcripts (Table 1). The distribution of transcript length is very similar between the four species (Figure 1), and in all cases a large portion of the assembly is contained within transcripts that are over 1000 bp in length. We have taken several approaches to evaluate the quality of each assembly and determine how comparable the four assemblies are. First, we identified which transcripts from three closely related species (*B.distachyon*, *O. sativa* and *T. aestivum*) share the greatest sequence similiarity with transcripts from the four *Lolium-Festuca* complex species. We then determined how much overlap there was between the transcript from our de-novo assemblies and the transcript from the related species. A high proportion of the transcripts can be aligned fully (100%) or almost fully (80%) to the transcripts from the related species (Table 2). The highest number of hits were found to the wheat gene set, the closest relative in this comparison. Secondly, we used the CEGMA pipeline [32] to evaluate the completeness of our assemblies. This is a tool that assesses the presence and coverage of a set of 248 extremely conserved core eukaryotic genes (CEGs). The tool is routinely used for evaluating genomic assemblies, however, it has also been used for evaluating transcriptome assemblies [33,34]. The percentage of complete CEGs ranged from 88.71 to 95.56, and the percentage of partially complete CEGs ranged from 94.76 to 97.58 (Table 3). The average number of orthologs per CEG and the % of detected CEGs that had more than 1 ortholog were similar across the four species. Our results point to transcriptome assemblies that reflect a representative portion of the transcriptome complexity, and are comparable between the four species.

**Table 1:** **Statistics of the filtered** ***de-novo*** **assemblies**

**Species**	**Number of sequences**	**Total nucleotide count**	**Max. transcript length**	**Average transcript length**	**N50**
*F. pratensis*	72,133	81,313,606	15,432	1,127	1,791
*L. multiflorum*	55,570	58,246,475	15,160	1,048	1,646
*L. m. westerwoldicum*	52,166	55,142,140	15,224	1,057	1,651
*L. temulentum*	59,309	67,863,492	15,295	1,144	1,802

**Table 2:** **Full length transcripts analysis**

**Species**	**Template protein dataset**	**100% coverage**	**> 80% coverage**	**> 20% coverage**
	Wheat	9,356	13,681	21,918
*F.pratensis*	Brachypodium	7,488	10,430	15,350
	Rice	6,355	9,391	13,360
	Wheat	8,320	12,016	19,749
*L.multiflorum*	Brachypodium	6,966	9,646	14,250
	Rice	5,895	8,657	12,518
	Wheat	8,352	12,088	19,615
*L.m. westerwoldicum*	Brachypodium	7,028	9,695	14,276
	Rice	5,905	8,720	12,495
	Wheat	9,063	13,103	20,642
*L. temulentum*	Brachypodium	7,543	10,481	15,118
	Rice	6,328	9,368	13,154

**Table 3:** **Results of CEGMA analysis**

**Out of 248**	***F. pratensis***	***L. multiflorum***	***L.m. westerwoldicum***	***L. temulentum***
% of fully represented	95.56	91.53	88.71	92.74
% of at least partially represented	97.58	95.56	94.76	97.58
average number of orthologs per CEG	2.56	2.56	2.37	2.4
% of detected CEGs with more than 1 ortholog	65.82	70.48	67.27	65.22

We predicted protein coding sequences from our transcriptome assemblies using Transdecoder [31], and the numbers of predicted proteins ranged between 30,182 and 39,981. We then looked at the percentage of proteins predicted from complete transcripts, that is having both the 3’ and 5’ UTRs present - their numbers ranged from 10,680 to 16,850. Pfam domains have been assigned for between 54.82 and 60.23% of the proteins for each species. Around 4% of the proteins were predicted to have signal peptides and around 15% to have transmembrane helices. The number of transcripts with GO terms assignment was between 54.8–60.8% (Table 4). A functional annotation report for each species is provided in Additional file 1: Table S1, Additional file 2: Table S2, Additional file 3: Table S3 and Additional file 4: Table S4.

**Table 4:** **Overview of functional annotation output of the four species transcriptomes**

**Species**	**Proteins predicted**	**Pfam**	**sprot**	**signalp**	**tmHMM**	**GO**
*F. pratensis*	39,981	21,921	21,653	1,622	5,630	21,928
*L. multiflorum*	30,940	18,464	18,386	1,400	4,609	18,569
*L. m. westerwoldicum*	30,182	18,179	18,170	1,339	4,703	18,349
*L. temulentum*	35,005	19,507	19,248	1,524	5,104	19,488

### Comparative gene family analysis

One way of understanding differences between related species on a genome-wide scale is to compare and find contrasts in the entire gene complement of each species. Best reciprocal BLAST hits between genes within a single species suggests the genes are paralogs. Best reciprocal BLAST hits between genes from different species suggests the genes are orthologs, and this strategy is widely used to generate orthologous pairs [35]. We used OrthoMCL [36] in order to compute orthologous clusters for all of our predicted proteins from the four species. We filtered proteins for the longest peptide predicted from a single representative transcript per locus, in order to avoid bias in the creation of the orthologous groups. We generated 15,930 clusters, assigning 57,822 (76,59%) to clusters of sizes from 2 to 176 proteins. The number of proteins contained in all clusters for each species varied between 14,161 and 14,835.

**Figure 2:**
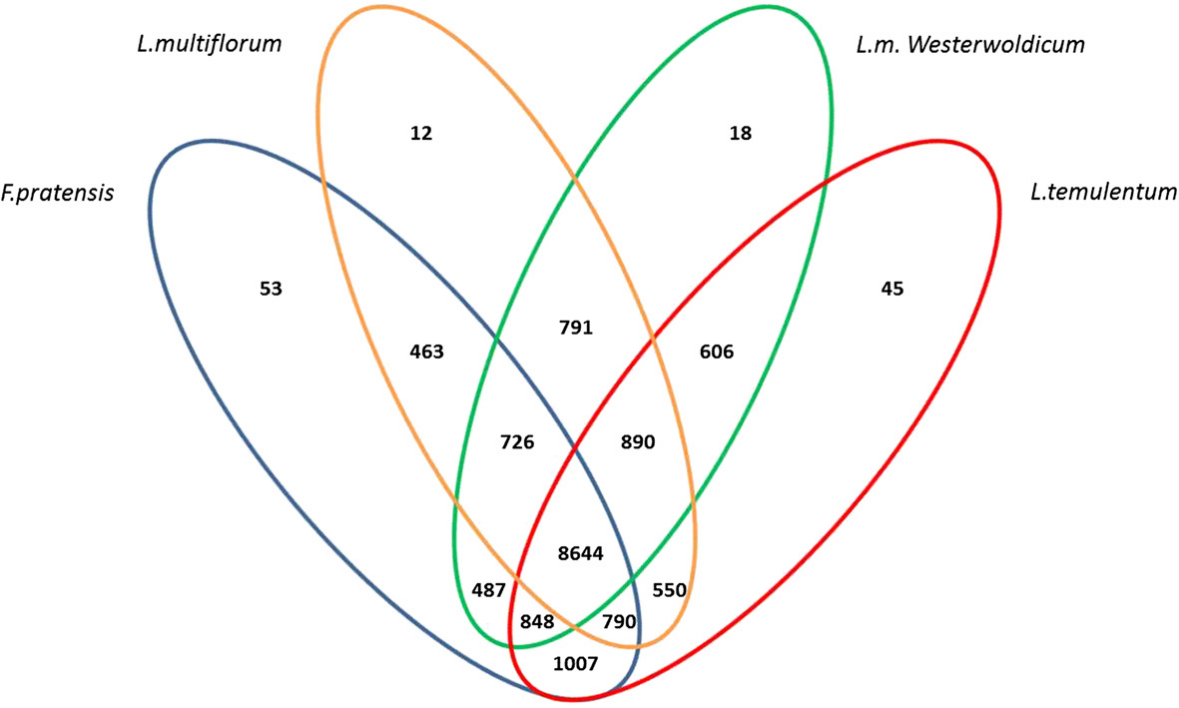
**Orthologous groups distribution.** The Venn diagram shows the distribution of shared and divergent orthologous groups from an OrthoMCL analysis of *Lolium-Festuca* complex proteomes, based on non-redundant dataset. The numbers in each division show the amount of groups for each combination.

Most of the proteins are found in clusters containing genes from at least two species, with 8,644 gene families shared between all four species (Figure 2). The number of unique (species-specific) clusters is relatively low, which is not surprising considering that the analyzed species are seperated by very small evolutionary distances. *L. multiflorum* and *L. m. westerwoldicum* have the smallest number of species - specific proteins, and many protein sequences that are shared only between these two. Again, this is not surprising because *L. m. westerwoldicum* is a ‘species’ derived from *L. multiflorum* through selective breeding for annuality [37]. Out of the gene families identified as unique, two predicted proteins from *F. pratensis* are showing high sequence identity with a ZCCT2-A2 VRN2 homologue from *T. urartu* [B8X8J1]. VRN2 has an important role in the vernalization/flowering pathway, by preventing the flowering of the plant unless it has experienced a period of cold temperatures and/or short days [38]. If there is a cold period, VRN2 becomes downregulated and allows the expression of the FT1 gene, which promotes flowering [39,40]. *F. pratensis* is a perennial species with predominantly a strong vernalization requirement [41]. All of the other species analyzed are of bi-annual or annual type and have a facultative (*L. multiflorum*) or no vernalization requirement (*L. m. westerwoldicum* and *L. temulentum* [42]). Samples for RNA-seq were taken from non vernalised plants, and it is therefore not surprising that VRN2 has been identified in the transcrtipome assembly of *F. pratensis*. The *Lolium* species without a vernalisation requirement do not have the VRN2 transcript present in their assemblies. A blastp alignment of the identified *Festuca* protein against the other transcriptomes revealed no significant hits. None of the original reads from the other species align back to the predicted VRN2 transcript, confiming that the VRN2 transcript is not present in the RNA-seq data sets of non-perennial species. We know from other studies that VRN2 is expressed in a *L. perenne* which does have a strong vernalization requirement [43]. The absence of VRN2 expression has been proven to enable FT induction and flowering in the closely related cereals [44,45]. Loss of function of VRN2 in wheat results in plants that do not require vernalization to flower, and it is the genetic locus responsible for distinguishing spring and winter wheat types [46]. Our results suggest that VRN2 is a key gene for differentiating vernalisation and non-vernalisation requiring species withinin the *Lolium-Festuca* complex. Other proteins identified as being species-unique included disease resistance proteins for *F. pratensis*, ABC transporter C for *L. multiflorum*, part of a ubiquitin ligase complex for *L. m. westerwoldicum* and ubiquin for *L. temulentum* (Table 5).

**Table 5:** **Annotation of the species-unique proteins identified**

**Species**	**Selected protein homologs**
	ZCCT2-A2 VRN2 homologue [*T. urartu*]
	Serine/threonine-protein kinase SMG1 [*A. tauschii*]
	serine/threonine-protein kinase GSO1 [*A. tauschii*]
	Hydroxyisourate hydrolase [*A. tauschii*]
	Disease resistance protein RPM1 [*T. urartu*]
	Putative disease resistance protein RGA4 [*T. urartu*]
	ABC transporter G family member 37 [*T. urartu*]
*F. pratensis*	60S ribosomal protein L28-1 [*A. tauschii*]
	Ribosomal L1 domain-containing protein 1 [*T. urartu*]
*L. multiflorum*	ABC transporter C family member 3 [*T. urartu*]
	Coatomer subunit beta’-2 [T. urartu]
	Kinesin-like protein KIF15 [*T. urartu*]
	Kinesin-like protein KIF15 [*A. tauschii*]
	GRF zinc finger family protein [*O. sativa* Japonica Group]
	DNA mismatch repair protein mutS [*A. tauschii*]
*L.m. westerwoldicum*	Splicing factor 3A subunit 3 [*T. urartu*]
	DNA mismatch repair protein Mlh1 [*A. tauschii*]
	ubiquitin [*S. aucuparia*]
	calcineurin B-like protein 4 [*T. aestivum*]
	fasciclin-like protein FLA14 [*T aestivum*]
	anthranilate N-benzoyltransferase protein 1 [*A. tauschii*]
	calcineurin B-like protein 4 [*T. aestivum*]
	cyclopropane-fatty-acyl-phospholipid synthase [*T. urartu*]
*L. temulentum*	B3 domain-containing protein [*A tauschii*]

#### Analysis of clusters with high and low sequence similarity

The average identity of sequences in the OrthoMCL groups indicates the level of similarity among proteins belonging to that group. The combined average sequence identity (referred to as %id) of all protein families was 91.61%. 747 families contained highly conserved proteins, and their %id was equal to 100. 2,056 families have a %id below 80%, constituting less conserved groups. Using the DAVID database [47] we have analyzed which functional annotation terms are overrepresented in the groups with different levels of percent sequence identity. GO Biological Process, INTERPROSCAN, and KEGG Pathway terms have been used for the annotation. Out of the proteins from groups having 100% identity 513 sequences could be matched in the DAVID database. They have been grouped into 45 clusters enriched for GO Biological Process terms. The most abundant classes of enriched terms include response to abiotic stress, ubiquitination, phosphorus metabolism, electron transport chain, protein localization, response to organic and hormone stimulus, positive regulation of transcription, carbohydrate metabolism, cell cycle, and meiotic cell cycle. Enriched KEGG pathway terms included purine and pirimidyne metabolism, pyruvate metabolism, glycolisis/gluconeogenesis, carbon fixation, biosynthesis of plant hormones, terpenoids, steroids and alkaloids, and citrate cycle. Enriched INTERPRO domains were related to ubiquitin, protein kinases, GTPases, ATPases, EF hands, and DNA/RNA helicases. Genes responsible for terms like basic metabolic processes related to biosynthesis and degradation, transcriptional and translational activity, protein synthesis and destination and signal transduction are amongst the most conserved in plants [48]. The same is true for genes involved in basic cell cycle machinery [49].

The families with a low %id represent proteins with less restrained sequence conservation, with possible multiple copies allowing for more relaxed selection. For the families having below 80%id we have identified 1,548 IDs using DAVID, which group into 90 clusters enriched for GO Biological Process terms. Clusters with the highest enrichment scores consisted of proteins related to phosphorylation, enzyme linked receptor protein signalling pathway, response to radiation, light and abiotic stimulus, protein ubiquitination, proteolysys and protein catabolic processes, response to organic and hormone stimuli, ion transport, root development, nucleotide metabolic processes and response to hormone stimulus. Three clusters were identified for enriched KEGG pathways, related to metabolism of methane, cyanoamino acid and glycine, serine and threonine, phenylopropandoid biosynthesis, and gluconeogenesis, biosynthesis of alkaloids and terpenoids. 64 clusters have been enriched for INTERPRO domains, with ten highest containing protein kinases, ABC transporters, ubiquitin, ATPases, zinc fingers, sulfphate ion transporters, DNA/RNA helicases, EF-hands, EGF-like domains, and PAS domains. Full overview of the GO Biological Process annotation is available in Additional file 5: Table S5 and Additional file 6: Table S6.

### Phylogenetic analysis based on orthologous gene families

The exact taxonomy of the *Lolium-Festuca* complex species is complicated and historically not completely agreed upon, with questions raised about the relationship between different *Loliums* as well as the origin of the species. The genus *Festuca* is considered to be ancestral to the genus *Lolium*, as it incorporates far more species and contains natural polyploids [4,13,50]. Evidence exists for both (i) the evolution of *Loliums* from a perennial *Festuca* subgenus *Schedonorus* ancestor [51], and (ii) a common ancestral form for both *Lolium* and *Festuca* [3,4]. Some reports are in favor of classifying the genus *Lolium* as part of the *Schedonorus* [52,53]. In general, the *Lolium* genus can be separated based on self-pollinating or out-pollinating behaviour. The most recent and complete analyses of the *Lolium-Festuca* complex reports the crown age of the *Lolium - Festuca* complex to be 8.97 +- 1.5 Ma. It also reports the *F. pratensis* to have originated in the Southwest Asia around 2 million years ago, and the *Loliums* to have first diversified in the eastern Mediterranean region around 4.1 Ma [54].

**Figure 3:**
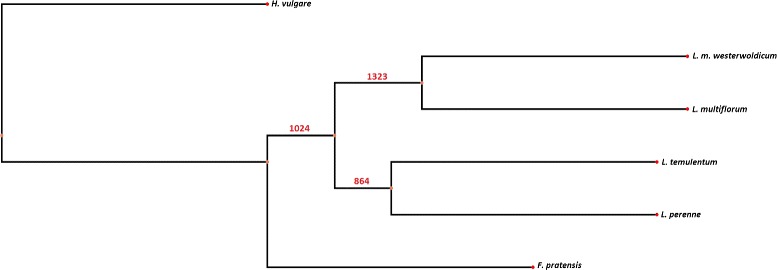
**Extended majority rule consensus tree.** A consensus phylogenetic tree, created from 4022 individual trees originating from OrthoMCL groups with one representative per specie. The numbers on the branches indicate the number of times the species have been partitioned into two sets.

In our study we performed the phylogenetic analysis using the orthologous groups identified by OrthoMCL. The clustering output was further filtered for conserved orthologous genes as a representative from each species - that is, having exactly one representative in the cluster for each of the species. 4022 groups fulfilled this criteria. Using these groups we inferred gene trees (using PAML tool) which were then clustered using the Phylip tool to infer a consensus tree (Figure 3). It is important to note that gene trees were calculated based solely on the gene-coding regions obtained from assembled transcriptome sequences only. The numbers on the branches indicate the number of times the species are partitioned into the two sets (out of 4022 groups). This means, that the branch topology has the highest support, or has been represented most commonly in the input trees. However, we find that a high proportion of the original trees have a different topology. This points to a different phylogenetic relationship depending on the group of orthologous proteins analyzed. These findings make sense in light of the fact that some of the species in the complex are interfertile. The fact that introgression of genes is possible within the complex has been utilized in breeding efforts as well as in research [55-57]. The genomes of modern grass species are a result of more complex evolutionary mechanisms, and reticulate evolution in the complex has been previously proposed [58]. The consensus tree (phylogeny) strongly corresponds to the possible phylogeny of the *Poaceae* family reported in [54].

### Genes under positive selection pressure in *Lolium* species compared to *F. pratensis*

**Figure 4:**
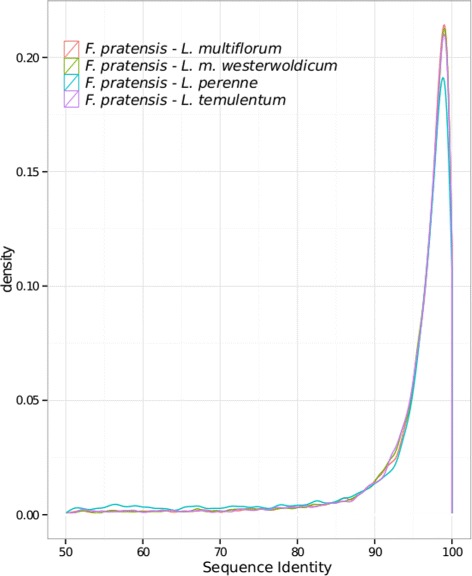
**Sequence identity distribution of pairwise comparisons.** The graph presents a distribution of protein identity between *F. pratensis* and the other *Lolium-Festuca* complex species used in pairwise BLAST comparisons. The kernel density plots are used here to view the distribution of a sequence identity. The X axis represents sequence identity (SqId) and the Y axis shows kernel density.

**Figure 5:**
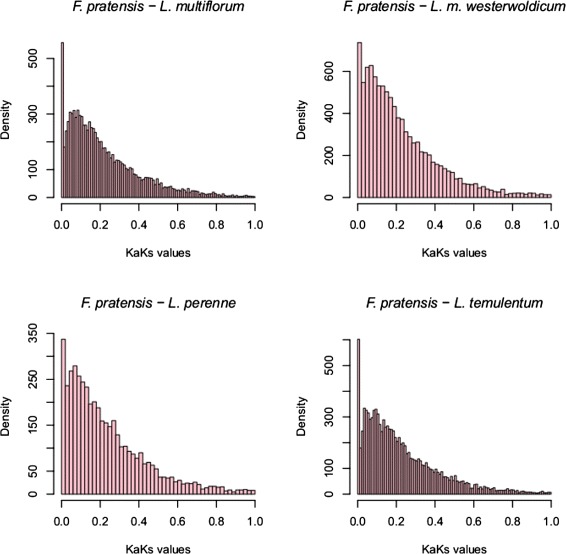
**Ka/Ks distribution.** The figure presents comparison of *F. pratensis* against *L. temulentum, L.m. westerwoldicum, L. multiflorum* and *L. perenne*. The frequency distributions of Ka/Ks rates (x-axis) shown here are based on protein and nucleotide alignments of orthologous genes.

We can identify two types of changes in the coding sequences - non synonymous (dN) substitutions, leading to change in the amino acid sequence, and synonymous (dS) substitutions, which are neutral for the amino acid sequence. The ratio of non-synonymous substitution rate (Ka) and synonymous substitution rate (Ks) is a parameter widely used to assess whether there is any directional selection acting on a protein coding gene. A ratio < 1 indicates that the protein is under purifying selection, whereas a ratio of > 1 is a good indication of positive selection pressure [59,60]. In such a closely related clade, only a small amount of genes can be expected to be responsible for phenotypical differences [61]. We undertook pairwise comparison of transcriptome datasets. Putative orthologous genes have been then classified according to the best bi-directional blast criteria (see Methods), and for every comparison we selected transcript pairs with a Ka/Ks ratio above 1. We have focused on comparing the *F. pratensis* with the other analyzed species, as it has various features that are important from an agricultural perspective, which include superior biotic and abiotic stress tolerance, good persistency and perenniality. The sequence identity distribution is very uniform among the pairwise comparison, with it’s peak around 95% (Figure 4). We focused our analysis towards genes involved in stress resistance, cell cycle and development related proteins, with the most relevant ones identified listed for every comparison. The overall distribution of Ka/Ks ratio of all pairwise comparisons is very similar, with almost every pair of proteins showing signs of purifying selection (Figure 5). The median ratio was very consistent, between 0.1741 for *L. multiflorum* and 0.1883 for *L. perenne*.

**Figure 6:**
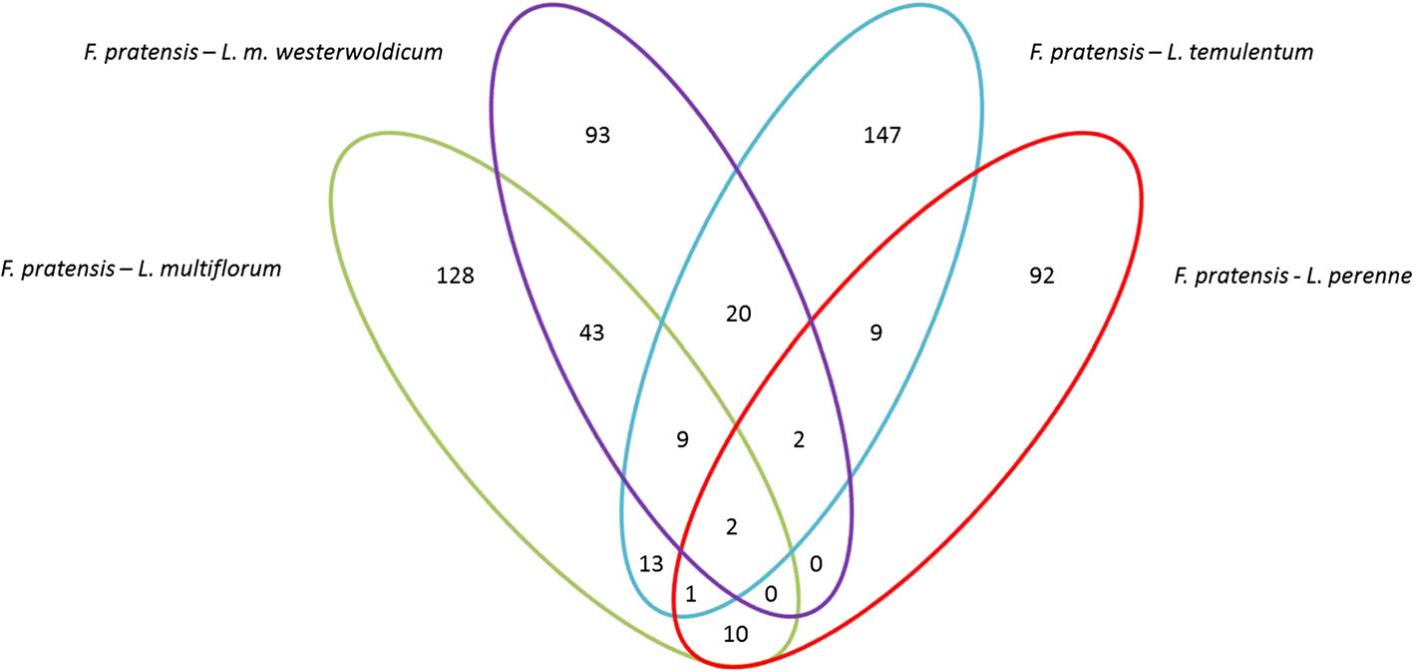
**Organisation of proteins under positive selection in** ***Festuca*** **to** ***Loliums*** **comparison.** The diagram show the number of proteins under positive selection between *Festuca* and analyzed *Lolium* species.

The number of orthologous pairs for which Ka and Ks could be calculated and was above 1 was equal to: 210 for *F. pratensis* and *L. multiflorum* (Additional file 7: Table S7), 177 for *F. pratensis* and *L. m. westerwoldicum* (Additional file 8: Table S8), 203 for *F. pratensis* and *L. temulentum* (Additional file 9: Table S9), 124 for *F. pratensis* and *L. perenne* (Additional file 10: Table S10). All of the pairs have been linked to their functional annotations. We have then categorized the transcript pairs that are under positive pressure in multiple comparisons, by checking how many *Festuca* identifiers are being shared between the pairwise comparisons (Figure 6). The majority of pairs have shown Ka/Ks values over 1 in only a single pair-wise comparison. However, there were three pairs shared in every analyzed comparison, and thus differentiating the *Festuca* from the *Lolium* species. The first was a homologue of *A. thaliana* ribosomal protein L4, one of the primary rRNA binding proteins [62], and the second was a UNC93-like protein 2, which is an integral component of the cell membrane [63]. In addition, in every pairwise comparison we detected a homologue of disease resistance protein RPM1, involved in plant defense against *P. syringae* in *A. thaliana* [64]. A homologue to disease resistance protein RPP13 conferring resistance to *Peronospora parasitica* in *A. thaliana* [65] has been identified in *L. perenne*, *L. multiflorum* and *L. temulentum* comparisons. Other proteins involved in plant-pathogen interaction, RPP8 and RPH8A, have been found in the *L. multiflorum* comparison. Different types of F-box proteins, which are mediating protein-protein interactions, were also abundant in every pairwise comparison.

Because *F. pratenesis* and *L. perenne* are perennial plants, and *L. multiflorum*, *L. m. westerwoldicum* and *L. temulentum* have a bi-annual or annual growth cycle, protein types present in every type of comparison except for *F. pratensis - L.perenne* have been closely investigated. One example of such proteins are cyclins, family of conserved proteins responsible for the control of cell-cycle progression [66]. Cyclin T1-1, has been identified in all comparisons except for the comparison with *L.perenne*. Other cyclins, T1-4 and T1-5, and Cyclin-dependent kinase F-4 have been identified in pairwise comparisons with *L. temulentum* E3 ubiquitin ligases have also been identified in every comparison apart from *L.perenne* - RNF128 in *L. multiflorum*, RFWD3 in *L. m. westerwoldicum* and RNF25 in *L. temulentum*. Additionally, multiple diverse transcription factors have been identified in non - *L.perenne* comparisons. These proteins constitute a group worth investigation of the perenniality/annuality trait genetic background.

When analyzing PFAM domains, the most abundant classes in every comparison were Leucine Rich Repeats, AAA domains and Tetratricopepdide repeats. All three of those protein domains can be found in proteins involved in diverse functions - such as protein-protein interactions, transcription factors, protein degradation and signal transduction. The full list of annotated proteins and PFAM domains is available in Additional file 11.

Apart from the pairwise comparisons of *Festuca* to *Lolium* species, we have also performed a comparison of *L. multiflorum* and *L. m. westerwoldicum*, assuming that a large amount of changes on the molecular level might have been caused by human influence [37]. It is an interesting comparison as *L. m. westerwoldicum* was developed by selecting *L. multiflorum* plants for annuality. A very high number of positively selected orthologous pairs - 235 - has been identified for these two species (Additional file 12: Table S11). As the main difference between the species is the strictly annual habit of *L. m. westerwoldicum*, apart from the basic metabolism and disease resistance we were also interested in proteins related to development and perenniality-annuality cycle. Annotations extracted from the previously created annotation files (Additional file 1: Table S1, Additional file 2: Table S2, Additional file 3: Table S3 and Additional file 4: Table S4) included multiple ubiquitin protein ligases as well as Cyclin-T1-1. Multiple disease resistance proteins have been identified: two RGA2 proteins, 1 RPM1 and one RPP13 protein. Among the pfam domain annotations we have found one that is related to seed dormancy control [PF14144.1], and two genes with a anaphase-promoting complex subunit [PF12861.2]. We have also identified multiple domains associated with sugar metabolism, such as fructose-1-6-bisphosphatase [PF00316.15], sugar efflux transporter for intercellular exchange [PF03083.11], MFS/sugar transport protein [PF13347.1] and sugar transporter [PF00083.19]. Another interesting category of domains included drought induced 19 protein (Di19) [PF05605.7] and Arabidopsis broad-spectrum mildew resistance protein [PF05659.6]. In spite of the extremely close phylogenetic distance, the amount and diversity of proteins under putative positive selection between those two species is very high, likely reflecting the intense selection pressure applied during the breeding of *L. m. westerwoldicum* from *L. multiflorum*.

Many of the enriched terms identified as being positively selected in this study share functions comparable to the ones in similar analyses [61,67,68]. Terms associated with protein kinases, protein phospthatases, transcription regulation and glycotransferases are linked to disease resistance [67], which are one of the fastest evolving and critical proteins in plant evolution. Terms related to stress response were present in almost every comparison, which is not suprising given the phenotypic background of the plants. The VRN2 gene has been identified as being important for determining spring or winter wheat varieties [46]. We have often observed terms related to reproductive structure development. Seeds and fruit size are one of the most distinct differences between wild and domesticated plants. *L. temulentum* is considered to be a mimic weed of wheat, and as such it has been involuntarily domesticated alongside that species [69]. Breeding of perennial grasses has a much shorter history, with the earliest records of it starting around 90 years ago [70]. However, given the intensity of modern breeding programs and the fact that *F. pratensis, L. multiflorum and L. m. westerwoldicum* plants used in our study are a result of a directed breeding effort, it might be worth investigating if some of the observed variation could be related to domestication like processes.

## Conclusions

This study presents the first de-novo transcriptome assemblies for four species from the *Lolium-Festuca* complex, and uses them to perform comparative transcritpomics. The orthologous genes between the species have a very high sequence similarities (91,61%), and the majority of gene families were shared for all of them. A consensus phylogenetic tree based on our large set of one-to-one orthologous genes is in agreement with the most recent study that was based on nuclear ribosomal intergenic spacer and plastid trnT-L and trnL-F regions. It is likely that the knowledge of the genomes will be largely transferable between species within the complex.

## Methods

### Sample collection

In order to capture a broad sequence diversity, we have chosen four *Lolium-Festuca* species that differ highly between each other with regard to phenotypic traits. The seeds were obtained from the breeding company DLF-Trifolium, and three of the species were commercial breeding varieties; *F. pratensis* - “Laura”, *L.multiflorum* “Lemtal”, *L. m. westerwoldicum* “Nerissa”. *L. temulentum* was a wild type. The seeds have been germinated and grown in the greenhouse under standard conditions. The RNA has been isolated on the 16th of April 2013 from mature leaf samples from single genotype of each of the four species using the RNeasy plant mini kit from Qiagen according to the manufacturers protocol.

### cDNA preparation and sequencing

cDNA library preparation has been done using the TruSeq kit, generating paired-end libraries with insert size of 300 bp. Sequencing has been carried out by the Beijing Genomics Institute, using Illumina Hi-Seq platform (91 bp paired-end sequencing), as well as the Ion Proton platform for a subset (25,5 milion reads) of *L. temulentum* sequences (91 bp single-end sequencing). The adapters were removed and reads were quality trimmed by BGI.

### Error correction

The original reads have been corrected for sequencing errors using the Allpaths-LG software (version 44837) built-in error correction tool [71], with default parameters. The tool is based on an algorithm eliminating exceptions from an overwhelming consensus read stack [30]. This process has reduced the total amount of paired-end reads by between 92.4 and 94.8%.

### Assembly

The samples from Illimuna and Ion Proton platforms have been merged and used together in the assembly. Trinity software [31] (version r2013_08_14) has been used for the generation of independent *de-novo* transcriptome assemblies, using the following parameters: –JM 20G –min_contig_ 200 –full_cleanup –min_kmer_cov 2. This has resulted a total number of 96,710 assembled transcripts for *F. pratensis*, 69,651 for *L.multiflorum*, 63,112 for *L. m. westerwoldicum* and 76,751 for *L. temulentum*. The reads have been mapped back to their assembly using RSEM (version 2013-02-16) [72] in order to filter out transcripts with low support.

### Protein prediction

Transdecoder (version r2013_08_14) [31] has been used to *de novo* predict putative coding regions and protein sequences using filtered transcripts as input. 39,981 proteins were predicted for *F. pratensis*, 30,940 for *L. multiflorum*, 30,182 for *L. m. westerwoldicum* and 35,005 for *L. temulentum.* CEGMA [73] pipeline was used to assess the completion of the transcriptome assemblies.

### Annotation

The trinotate (release 2014.07.08) [31] pipeline was used for the annotation of the protein dataset. Both transcripts and proteins have been aligned using blast software [74] (version 2.2.28+), blastx and blastp respectively, using swissprot protein database (release 09_07_2014) as the target. HMMER [75] (version 3.1b1) with Pfam-A database [76] (version 27.0) has been used to identify protein domains, signalP [77] (version 4.1) was used to predict signal peptides and tmHMM [78] (version 2.0c) was used to predict transmembrane helices. The resulting information has been loaded into SQLite database and wrapped up by the Trinotate report script, creating a comprehensive annotation.

### Validation of assembly completion by CEGMA

CEGMA software [73] (version 2.4.010312) has been used to assess the completion of the transcript dataset. The software was run with default parameters with the included reference dataset.

### Full-length transcript analysis

We have used high confidence protein datasets from *T. aestivum*, *B. distachyon* and *O. sativa*, downloaded from MIPS PlantsDB database (30.07.2014) [79]. NCBI blastx was used (-evalue 1e-20 -max_target_seqs 1 -outfmt 6) to align each of our four filtered transcriptome assemblies to each of the protein databases separately. Afterwards we have used the analyze_blastPlus_topHit_coverage.pl script from the Trinity package to identify unique top matching proteins that align across certain length thresholds of the template sequence.

### Orthologous group assignment

Predicted protein sequences have been clustered into orthologous groups using the OrthoMCL software [36]. The input protein dataset predicted by transdecoder has been filtered to contain only the proteins predicted from the longest, unique transcript splice variants, giving 19,863, 17,718, 18,095, and 19,817 proteins, respectively. A custom perl script was used in order to get information about the number of clusters shared. A set of custom python scripts has been used to get information about groups with over- and underrepresented sequences and assesing a group wide pfam domain classification. Sequences from the species-unique groups have been aligned online with the NCBI protein database for manual functional annotation. Sequences having %id hits below 40 have been discarded. Sequences having hits to putative or predicted proteins without any assigned function have not been considered for further analysis.

### Identification of putative orthologs

For the identification of putative orthologs between two species, we applied bi-directional blastp [80] where two sequences are considered as orthologous if they satisfy a sequence identity cut-off over the length of amino acids > 30 [81]. We have used the *F. pratensis* protein dataset predicted earlier for the identification of proteins under positive selection.

### Estimation of synonymous and non-synonymous substitution rate

Orthologous gene pairs were aligned using CLUSTALW [82]. The maximum likelihood estimation of synonymous (Ks) and non-synonymous (Ka) substitution rate was estimated using the yn00 module of the PAML4 suite [83].

### Analysis of evolutionary conserved and relaxed groups

For every sequence pair showing a Ka/Ks ratio higher than 1 and below 10, a representative sequence was chosen. It was belonging to the *F. pratensis* for all but one case. *L. multiflorum* representative was chosen for the *L. multiflorum* and *L. m. westerwoldicum* pair. The choice of one of the two representatives did not change the final outcome of the analysis. Matching uniprot identifiers have been identified by aligning the representative sequences with *A. thaliana* uniprot sequences (accessed 16.09.2014). The results have been filtered to contain only hits to plant species. The uniprot identifiers have then been used af input for DAVID. Clustering was done using the default parameters, with the KEGG pathway, INTERPRO domain and Biological Process GO terms used for annotation and *A. thaliana* sequences as a background for enrichment study.

### Phylogenetic analysis

OrthoMCL clusters have been filtered in search of clusters containing a single representative from each species. 4022 groups have been selected and used to infer gene trees using PAML4 suite [83]. The resulting trees have been which were then clustered using the consensus tree program, version 3.69 of the Phylip package [84] to infer a consensus tree.

### Availability of supporting data

The error corrected transcriptome reads have been deposited in the SRA database under the following accession numbers: SRR1648382 (*F.pratensis*), SRR1648406 (*L. multiflorum*), SRR1648407 (*L.m. westerwoldicum*), SRR1648408, SRR1648409 and SRR1648494 (*L. temulentum*). The Transcriptome Shotgun Assembly project has been deposited at DDBJ/EMBL/GenBank under the accession numbers GBXZ00000000 (*F.pratensis*), GBXX00000000 (*L. multiflorum*), GBXY00000000 (*L.m. westerwoldicum*), and GBXW00000000 (*L. temulentum*). The versions described in this paper are the first versions, GBXZ01000000, GBXX01000000, GBXY01000000, and GBXW01000000 respectively.

## Additional files

Additional file 1
**Table S1.** Trinotate annotation report for *F. pratensis*.

Additional file 2
**Table S2.** Trinotate annotation report for *L. multiflorum*.

Additional file 3
**Table S3.** Trinotate annotation report for *L.m. westerwoldicum*.

Additional file 4
**Table S4.** Trinotate annotation report for *L. temulentum*.

Additional file 5
**Table S5.** Biological Process Annotation of 100% id groups.

Additional file 6
**Table S6.** GO Biological Process Annotation of >80% id groups.

Additional file 7
**Table S7.** Annotations of proteins under putative positive selection in pairwise comparisons: *F. pratensis - L. multiflorum*.

Additional file 8
**Table S8.** Annotations of proteins under putative positive selection in pairwise comparisons: *F. pratensis - L.m. westerwoldicum*.

Additional file 9
**Table S9.** Annotations of proteins under putative positive selection in pairwise comparisons: *F. pratensis - L.temulentum*.

Additional file 10
**Table S10.** Annotations of proteins under putative positive selection in pairwise comparisons: *F. pratensis - L.perenne*.

Additional file 11
**Number of orthologous protein pairs identified in** ***H.vulgare*** **vs** ***Lolium-Festuca*** **complex species using a range of %identity thresholds.**

Additional file 12
**Table S11.** Annotations of proteins under putative positive selection in pairwise comparisons: *L. multiflorum - L.m. westerwoldicum*.
