# Supplementary material for: Comparative transcriptome analysis within the Lolium/Festuca species complex reveals high sequence conservation
Source: BMC Genomics. 2015 Mar 28;16(1):249. doi: 10.1186/s12864-015-1447-y (PMC4389671; doi:10.1186/s12864-015-1447-y)
Supplement: Additional file 11 — Number of orthologous protein pairs identified in H.vulgare vs Lolium-Festuca complex species using a range of %identity thresholds. [file 12864_2015_1447_MOESM11_ESM.docx]

| seq_id | hv_lau | hv_lem | hv_ner | hv_per | hv_tem |
| --- | --- | --- | --- | --- | --- |
| 50 | 7786 | 7294 | 7457 | 4871 | 8021 |
| 72 | 7930 | 7503 | 7639 | 4814 | 8149 |
| 75 | 7720 | 7301 | 7442 | 4670 | 7907 |
| 80 | 7036 | 6671 | 6806 | 4250 | 7159 |
| 85 | 5705 | 5465 | 5536 | 3485 | 5810 |
| 90 | 3550 | 3454 | 3485 | 2211 | 3596 |
| 95 | 1086 | 1054 | 1057 | 702 | 1089 |

Table S1. Number of orthologous genes found using different sequence identity thresholds between *H. vulgare* and *Lolium-Festuca* complex species.

Figure S1. Graphical representation of Table S1. The x-axis represents the sequence identity threshold; the y-axis represents the number of orthologous pair identified.
